# Predicting 2-Year Early Allograft Failure After Kidney Transplant: A Perioperative Risk Model from the MOVER Cohort

**DOI:** 10.3390/healthcare14030341

**Published:** 2026-01-29

**Authors:** Thomas Renfrew, Peyton J. Murin, Madison L. Schanz, Hamed Sadeghipour, Yuri Chaves Martins

**Affiliations:** 1 School of Medicine, Saint Louis University, St. Louis, MO 63104, USA; thomas.renfrew@health.slu.edu (T.R.); madison.schanz@health.slu.edu (M.L.S.); 2 Department of Neurology, School of Medicine, Saint Louis University, St. Louis, MO 63104, USA; peyton.murin@health.slu.edu; 3 Department of Anesthesiology, School of Medicine, Saint Louis University, St. Louis, MO 63110, USA; hamed.sadeghipour@health.slu.edu

**Keywords:** allograft failure, anemia, clonidine, kidney transplantation, obesity, perioperative risk model, thrombocytopenia

## Abstract

Background: Allograft failure after kidney transplantation remains common despite improving long-term outcomes and persistent organ scarcity. We aimed to develop and internally validate a perioperative risk model for kidney allograft failure within 2 years of transplantation. Methods: We conducted a single-center retrospective cohort study using the Medical Informatics Operating Room Vitals and Events Repository. Adult patients (≥18 years) undergoing kidney transplantation between January 2018 and July 2023 with at least 2 years of follow-up were included. The primary outcome was allograft failure within 2 years, defined as return to dialysis, pre-emptive re-transplantation, or death. Candidate predictors included demographic characteristics, comorbidities, preoperative laboratory values, and intraoperative variables. After univariate screening and variable selection with LASSO-penalized regression, we estimated relative risks using modified Poisson regression and assessed internal validity with 200 bootstrap resamples. Results: Among 319 recipients, 53 (16.6%) experienced early allograft failure. In the final multivariable model, obesity (relative risk [RR] 4.76; bootstrap 95% CI 2.88–9.31) and thrombocytopenia (RR 1.96; bootstrap 95% CI 1.18–3.38) were independently associated with increased risk. Anemia (RR 0.22; bootstrap 95% CI 0.13–0.37), preoperative clonidine use (RR 0.33; bootstrap 95% CI 0.00–0.85), and female sex (RR 0.55; bootstrap 95% CI 0.26–0.83) were associated with reduced risk. Model performance was modest (pseudo-R^2^ 0.21) but identified clinically distinct risk strata. Conclusions: A five-variable perioperative model based on obesity, thrombocytopenia, anemia, preoperative clonidine use, and female sex identified kidney transplant recipients at differing risk of allograft failure within 2 years. These associations highlight potentially modifiable targets that warrant further study and external validation before clinical use.

## 1. Introduction

Kidney transplantation is the preferred therapy for kidney failure [1]. Due to advances in the prevention and management of hyperacute and acute allograft rejection, kidney transplantation has achieved gains in allograft longevity over the past two decades [2]. However, access to transplant is still constrained by a persistent shortage of donor organs, leaving many eligible patients on dialysis for years [3,4]. In this setting, there is still a growing need for perioperative tools that can anticipate early allograft failure and support targeted interventions. International societies have therefore called for robust predictors of outcomes that can guide monitoring and individualized therapy and that can also serve as surrogate endpoints to accelerate clinical trials [5]. In addition, because allograft loss is multifactorial, approaches that combine complementary risk factors into multivariable prognostic scores consistently outperform reliance on single markers [6].

Perioperative care already follows multidisciplinary, enhanced recovery pathways [7], incorporating preoperative comorbidities, dialysis status, immunosuppression strategy [8], anesthetic management [9], and real-time assessment of perfusion and volume status [10,11]. Yet guidance often hinges on isolated parameters such as mean arterial pressure targets or fluid totals, and the supporting evidence is mixed. For example, some studies link a mean arterial pressure near 75 mmHg to reduced delayed allograft function, but the interplay of vasopressors, adherence to targets, and fluid strategy complicates interpretation [12,13]. These inconsistencies highlight the need for integrated, data-driven prediction rather than single-metric heuristics [13].

We therefore sought to develop and internally validate a parsimonious perioperative risk model of kidney allograft failure within 2 years of transplantation using routinely collected perioperative data from the Medical Informatics Operating Room Vitals and Events Repository (MOVER) cohort. Our aims were to quantify the discrimination and calibration of a multivariable prediction model, to identify perioperative features associated with <2-year allograft failure while acknowledging that associations do not imply causation, and to illustrate how such a model could support real-time risk stratification to complement existing clinical pathways and inform postoperative monitoring and resource allocation.

## 2. Materials and Methods

### 2.1. Study Design and Oversight

This retrospective cohort study was conducted using de-identified perioperative data from kidney transplant recipients at a single academic medical center. The study followed the Transparent Reporting of a Multivariable Prediction Model for Individual Prognosis or Diagnosis (TRIPOD) reporting standards for development of a multivariable prediction model. Since the MOVER database is de-identified in compliance with the Health Insurance Portability and Accountability Act (HIPAA) Privacy Rule, individual patient informed consent was not sought, in accordance with institutional policy for analyses of de-identified data.

### 2.2. Data Source and Population

**Figure 1:**
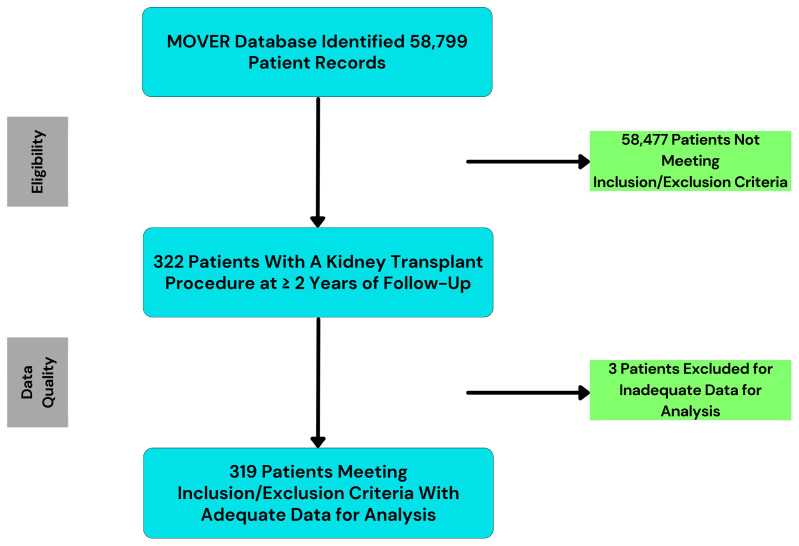
The Medical Informatics Operating Room Vitals and Events Repository (MOVER) dataset contained 58,799 patient records, including 322 patients with a kidney transplant procedure and ≥2 years of follow up. Of these, 319 patients met the inclusion criteria.

The authors accessed the dataset through a data use agreement with the University of California, Irvine Medical Center. Information was drawn from the MOVER database [14], which includes de-identified electronic health records for 58,799 adult surgical patients. The MOVER database was queried for patients who underwent kidney transplant surgery between January 2018 and July 2023 to ensure complete follow-up within the available dataset. Inclusion criteria: kidney transplant surgery and ≥2 years of follow up post-transplant. Exclusion Criteria: multi-organ transplant (4 patients) and <2 years of follow-up post-transplant (15 patients). We confirmed at least 2 years of follow-up by including only procedures that occurred ≥2 years before the data censoring date. All deaths within 2 years post-transplant were counted as events regardless of graft function. In total, 319 patient records were included in the study. The primary outcome was <2-year allograft failure, which was defined as return to dialysis, pre-emptive kidney re-transplantation, or death within 2 years of transplant. Figure 1 depicts the sampling process.

### 2.3. Predictors and Preprocessing

Candidate predictors were derived from current procedural terminology (CPT) codes, international classification of diseases (ICD)-9-CM codes, patient demographics, the American Society of Anesthesiologists (ASA) physical status score, past medical history, family history, anesthesia type, donor status (living vs. deceased), and perioperative clinical data. Perioperative hemodynamics and ventilator parameters were summarized into scalar features extracted from the intraoperative record (e.g., lowest mean arterial pressure, duration of mean arterial pressure < 65 mmHg, peak airway pressure), and perioperative medication exposures were encoded as binary indicators. A detailed description of all variables, their operational definitions, and their encoding is provided in Supplementary Table S1. Continuous predictors were rescaled to clinically meaningful increments (e.g., mean arterial pressure per 10 mmHg) to improve interpretability of relative risks. Between-group comparisons used Fisher’s exact test for categorical variables and the Mann–Whitney U test for continuous or ordinal variables.

### 2.4. Missing Data

Missingness patterns were examined before imputation. Overall missingness was low (2.19% of all predictor values). Continuous variables were imputed using mean imputation (SimpleImputer, scikit-learn 1.6.1). Although more complex imputation methods can be advantageous, the low level of missing data and the inability to model missingness mechanisms in a de-identified dataset supported the use of mean imputation for simplicity and transparency. We acknowledge that mean imputation does not account for uncertainty and may introduce bias if data are not missing completely at random, but it was considered a pragmatic approach for this dataset.

### 2.5. Feature Selection and Dimensionality Reduction

Given the modest event rate and to preserve adequate events per variable, feature selection was performed exclusively within the training dataset in a sequential, pre-specified manner:

1. Univariate Screening: Each predictor underwent univariate logistic regression with allograft failure as the dependent variable. Predictors with *p* < 0.25 were retained.
2. Correlation Filtering: Among retained variables, pairwise Pearson correlations were examined. Predictors with |r| > 0.80 were excluded to reduce multicollinearity.
3. Penalized Regression Screening: Remaining predictors were evaluated using LASSO-penalized logistic regression with 5-fold cross-validation. Predictors with non-zero coefficients were selected. The five predictors with the largest absolute coefficients were retained to maintain approximately 10 events per variable (53 events, 5 predictors).

### 2.6. Model Development

The risk of allograft failure was estimated using a modified Poisson regression with a log link and robust (HC3) standard errors. This approach provides stable and interpretable relative risks (RRs) for binary outcomes while avoiding the convergence issues associated with log-binomial models [15]. All models were fit using generalized linear models (GLM) with a Poisson family and log link. Predictors were entered linearly without interaction terms. Model discrimination was assessed descriptively using deviance, Pearson χ^2^, and pseudo-R^2^ (McFadden/CS) and visual inspection of predicted risk distributions. No recalibration or shrinkage procedures were applied.

### 2.7. Internal Validation

Internal validation was performed using nonparametric bootstrap resampling. A total of 200 bootstrap samples were drawn with replacement from the training dataset. In each bootstrap replicate, the full modified Poisson model was refit, and the distribution of exponentiated coefficients was used to derive bootstrap 95% confidence intervals (CIs) for each predictor.

### 2.8. Software

All analysis was performed using Python 3.11 (Python Software Foundation, Wilmington, DE, USA). The following add-ons were used for analysis: pandas [16], numpy [17], patsy [18], statsmodel.api [19], sklearn.model_selection, sklearn.preprocessing, sklearn.pipeline, sklearn.linear_model, sklearn.impute, and sklearn.utils [20].

## 3. Results

### 3.1. Cohort Characteristics

The study cohort consisted of 319 patients, of which 53 (16.6%) had allograft failure within two years. Only two patients were classified as allograft failure due to death. As expected, 100% of the patients had a diagnosis of end-stage renal disease, were dependent on dialysis, and were admitted to the ICU after their procedure (Table S2). There was no significant difference in age (51.04 ± 13.64 versus 49.17 ± 13.72, *p* = 0.36) or biological sex (29.6% versus 39.6%, *p* = 0.22) between patients with or without allograft failure (Table 1). Compared with those without <2-year allograft failure, patients with allograft failure had higher rates of obesity (*p* < 0.0001) and thrombocytopenia (*p* = 0.0013) and lower rates of coded anemia (*p* < 0.0001), and coded anemia in chronic kidney disease (*p* = 0.0218) (Table 1); other comorbidities and intraoperative parameters were broadly similar (Table S2). Several intraoperative medication exposures, including IV dextrose 50% (*p* < 0.0001), glucagon HCl 1 mg (*p* = 0.0002), acetaminophen (*p* = 0.0041), and midazolam (*p* = 0.0428), significantly differed among patients with early allograft failure (Table 1). These medications likely reflect perioperative metabolic or hemodynamic instability rather than primary causal risk factors. A complete univariate analysis of all candidate predictors is presented in Table S2.

### 3.2. Poisson Regression

The model converged within six iterations. Deviance was low at 0.36 with a Pearson χ^2^ of 0.79, suggesting no evidence of over-dispersion. The model displayed moderate explanatory power with a McFadden/CS pseudo-R^2^ of 0.21. Intraoperative medications significant in univariate analysis were excluded during feature selection because they were likely proxy markers of metabolic or hemodynamic instability, were collinear with other variables, or did not retain independent signal during penalized regression.

**Figure 2:**
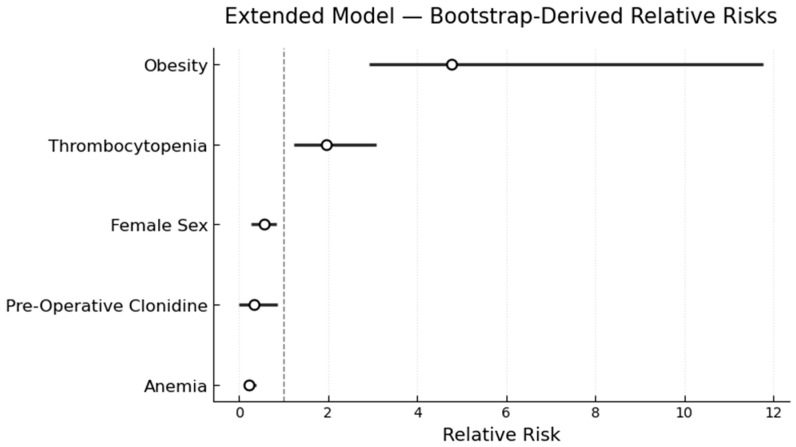
Forest plot illustrating the relative risk (RR, open circles) and 95% confidence interval (CI, horizontal lines) for five factors included in the Poisson regression model. Obesity (RR 4.76; bootstrap 95% CI 2.88–9.31) and thrombocytopenia (RR: 1.96; bootstrap 95% CI 1.18–3.38) conveyed an increased risk of allograft failure. Anemia (RR 0.22; bootstrap 95% CI 0.13–0.837), preoperative Clonidine (RR 0.33; bootstrap 95% CI 0.00–0.85), and female sex (RR 0.55; bootstrap 95% CI 0.26–0.83) conveyed a decreased risk. Vertical dashed line indicates RR equal to 1.0.

In a modified Poisson regression model with robust standard errors, obesity and thrombocytopenia were independently associated with an increased risk of allograft failure. Recipients with a medical history of obesity had a ~4.5-fold higher risk of allograft failure compared with non-obese recipients (RR 4.76; bootstrap 95% CI 2.88–9.31). Amongst remaining variables, thrombocytopenia (RR: 1.96; bootstrap 95% CI 1.18–3.38) was the only one associated with increased risk of allograft failure. Anemia was paradoxically associated with a lower risk of allograft failure (RR 0.22; bootstrap 95% CI 0.13–0.837). Preoperative Clonidine (RR 0.33; bootstrap 95% CI 0.00–0.85) and female sex (RR 0.55; bootstrap 95% CI 0.26–0.83) both conveyed decreased risk of allograft failure. (Figure 2). The model demonstrated a pseudo-R^2^ of 0.21, consistent with modest but clinically relevant discrimination.

## 4. Discussion

Our analysis identified obesity and thrombocytopenia as significant predictors of <2-year kidney allograft failure, whereas a history of anemia and pre-operative clonidine use were unexpectedly associated with lower failure risk. These findings offer a novel perioperative risk profile that both aligns with and diverges from prior literature on transplant outcomes.

The strong association between recipient obesity and early allograft loss in our cohort (RR ~ 4.5) is generally concordant with previous reports [21,22]. A large UK prospective study likewise found that obesity more than doubled the hazard of allograft failure among deceased donor kidney transplants [21]. Similarly, a paired-kidney analysis from the Australia and New Zealand Dialysis and Transplant Registry showed obese recipients had a higher risk of delayed allograft function and a 25% increased hazard of death-censored allograft loss compared to non-obese recipients [22]. Mechanistically, obesity is linked to greater surgical complexity and postoperative complications. Obese transplant recipients experience higher rates of wound infection, fascial dehiscence, and lymphocele formation, as well as prolonged hospitalizations [23]. Notably, obesity is an independent predictor of delayed allograft function, reflecting the vulnerability of the allograft to ischemia–reperfusion injury in the setting of adiposity [23,24]. Perioperative challenges combined with comorbidities such as hypertension, diabetes, and cardiovascular disease likely contribute to the inferior allograft survival observed in obese patients [21,22]. Therefore, our findings reinforce that optimizing body mass index prior to transplant or closely managing obese recipients postoperatively may improve outcomes. It is worth noting that some analyses have found obesity’s impact on long-term allograft survival diminishes after adjusting for co-variables like diabetes and donor quality [23]. In our study’s 2-year horizon, however, the effect of obesity was pronounced, underscoring the critical early period where technical and cardiovascular complications can precipitate allograft loss.

Past medical history of thrombocytopenia emerged as a novel predictor of <2-year allograft failure in our model (RR ~ 2.1). While thrombocytopenia is not traditionally highlighted in transplant risk scores, this finding is biologically plausible and supported by related literature [25,26,27]. Post-transplant thrombocytopenia often signals a coagulopathic state or severe illness (e.g., systemic infection or thrombotic microangiopathy), which can lead to bleeding or allograft thrombosis [25,26]. In fact, a recent study reported that ~9% of kidney recipients developed post-transplant thrombocytopenia (platelets < 100 × 10^9^/L after the first month), and one-third of these patients suffered significant bleeding complications [25]. In addition, this cohort found that over half of patients with post-transplant thrombocytopenia went on to experience allograft loss or death during follow-up [25]. These data suggest that thrombocytopenia serves as a proxy for events (like hemorrhage or disseminated intravascular coagulation) that imperil the allograft. For example, de novo thrombotic microangiopathy in the allograft typically presents with microangiopathic hemolysis and thrombocytopenia and is notoriously associated with allograft failure rates up to ~40% within 2 years [27]. In our cohort, detailed causes of thrombocytopenia were unavailable, but it is likely that patients flagged with this risk factor had underlying conditions (coagulopathy, platelet consumption, or marrow suppression) that led to perioperative hemorrhage or allograft hypoperfusion. This aligns with prior observations that severe post-transplant bleeding is a significant risk factor for allograft loss [25].

One unexpected result was that a history of anemia was associated with lower risk of early allograft failure in our model (RR ~ 0.29), despite univariate analysis showing higher anemia prevalence among those who experienced failure. This counter-intuitive finding contrasts with the bulk of transplant literature [28,29,30]. Generally, anemia in kidney transplant recipients has been linked with worse outcomes [30]. Recipients who are anemic in the early post-transplant period have significantly higher acute rejection rates, cardiovascular complications, risks of allograft failure, and mortality over 4 years [28,29]. Moreover, anemia is a well-known contributor to left ventricular hypertrophy and heart failure in patients with chronic kidney disease, which can increase perioperative cardiac risk [28]. Why, then, did our multivariable model yield a protective coefficient for anemia? We suspect this reflects residual confounding or collider bias rather than a true protective effect. Furthermore, we highlight the importance of this result not being interpreted as causal or clinically protective, as multiple factors are associated with development and severity of anemia in patients with end-stage renal disease [31]. In addition, patients without an anemia diagnosis could include those who maintained higher hemoglobin via frequent blood transfusions, whereas patients labeled with “anemia of chronic kidney disease” may have been managed instead with erythropoietin-stimulating agents. Furthermore, blood transfusions are a double-edged sword in transplant: while they treat anemia, they expose the recipient to foreign HLA antigens carried on donor leukocytes and platelets, potentially inducing allo-sensitization [32]. Sensitized patients have a higher burden of donor-specific antibodies and risk of rejection, which could compromise allograft survival. It is conceivable that in our cohort the “non-anemic” group harbored more heavily transfused (and thus sensitized) individuals, whereas anemic chronic kidney disease patients on medical therapy avoided some of this risk. This could create a spurious association where anemia appears protective after adjusting for other factors. Another possibility is that anemia in our data was an indicator of chronic stable disease (managed anemia) as opposed to acute illness. Given these possibilities and the contradiction with prior literature, the apparent protective association of anemia should not be interpreted causally. In addition, future analyses with granular data on transfusion history and antibody profiles could elucidate this relationship further.

Interestingly, female patients were less likely to have allograft failure than their male counterparts. This is not an entirely unsurprising finding, as prior literature has noted female patients are less likely to be waitlisted and receive a kidney transplant [33]. Thus, the female candidates who are waitlisted and receive a kidney are possibly better transplant candidates. This is supported by prior literature showing higher rates of kidney transplant failure in male patients [34]. However, the literature remains heterogenous, with some studies showing more favorable outcomes in men [35] or no difference [36]. Our findings may therefore reflect selection effects rather than inherent biological protection.

Among medications, clonidine use was associated with a protective effect in renal transplant patients. To the best of our knowledge, this is the first report specifically linking clonidine exposure with improved renal transplant outcomes. This observation is biologically plausible. Clonidine’s α2-adrenergic agonism can improve renal hemodynamics and blunt the perioperative catecholamine surge [37], and a prior randomized trial in cardiac surgery demonstrated that preoperative clonidine preserved postoperative GFR compared with placebo [38]. In kidney transplantation itself, perioperative dexmedetomidine, another α2-agonist, has been associated with lower rates of delayed graft function, early rejection, and postoperative complications, supporting the concept that α2-mediated sympatholysis during transplant may be protective [39]. Conversely, large perioperative trials such as POISE-2 did not show a reduction in AKI with clonidine and highlighted an increased risk of clinically important hypotension, which in turn was associated with higher AKI rates, underscoring that any renal benefit of clonidine is likely contingent on careful hemodynamic management [40]. In addition, clonidine’s analgesic properties may reduce perioperative opioid requirements, and higher opioid exposure has been linked to worse outcomes after renal transplantation [41]. Thus, our findings may reflect a combination of improved renal hemodynamics, modulation of ischemia–reperfusion and inflammatory responses, and indirect benefit through opioid-sparing analgesia. However, we find it important to note that this effect may have been in large part mediated by confounding by indication, especially given the observational nature of our data and the mixed results of large perioperative trials.

Unlike traditional prognostic models for kidney transplant outcomes, which emphasize donor characteristics and immunologic factors, our perioperative risk model focuses on immediate, modifiable clinical parameters [42]. For example, the iBox scoring system and other published risk scores incorporate variables such as donor quality, HLA mismatch, and serum creatinine trajectory to predict long-term allograft loss [43]. These tools overlook intraoperative or immediate postoperative factors [42]. Our findings suggest that perioperative physiology can substantially influence early outcomes and could complement existing risk stratification. Notably, a recent machine-learning study identified features like recipient comorbidities and hemodynamics as important for allograft survival prediction, aligning with our approach of integrating real-time surgical data rather than relying solely on baseline transplant metrics [13]. By developing a simple, parsimonious risk score from routinely collected intraoperative data, we contribute a novel tool that could be used alongside immunological risk assessment. This is especially pertinent for guiding early post-transplant care. For example, a patient flagged as high-risk by our model might benefit from closer hemodynamic monitoring, proactive ICU support, or early biopsy to check for treatable issues, even if their immunologic risk profile is low.

Several limitations temper the interpretation of our findings. First, this was a single-center, retrospective study using de-identified electronic health record data. The sample size (319 patients, 53 events) is modest for prediction modeling, raising the possibility of overfitting and wide confidence intervals for some predictors. In addition, our sequential univariate screening and LASSO selection pipeline, while necessary to preserve an adequate events/variable ratio, may have introduced selection bias and optimistic estimates of predictor performance. Indeed, the paradoxical effect of anemia may reflect such complexities. An internal bootstrap validation was performed, but we did not externally validate the model on an independent cohort—a necessary step before broader clinical application. Second, our definition of variables relied on EHR documentation and ICD coding, which may introduce misclassification. “Thrombocytopenia” and “acidosis” were recorded as binary features, but we lacked granular data (exact platelet counts, pH or bicarbonate levels) to set standardized thresholds. It is possible that these terms captured only more severe cases, and milder degrees went unrecognized. Third, we acknowledge that immunologic factors (donor-specific antibody levels, HLA mismatches) and donor organ quality (e.g., kidney donor profile index, cold ischemia time) were not explicitly included in our perioperative model. These factors unquestionably influence allograft outcomes [21], especially beyond the immediate postoperative period. Their omission could bias our model or limit its generalizability, though it also demonstrates the incremental value of perioperative data. Fourth, the outcome of “allograft failure” was defined to include death with function; while this is a common composite in transplant studies, it means that two events were primarily patient mortality. Thus, our risk factors (obesity and patient sex in particular) may also be capturing risk of early patient death in addition to allograft non-function. Fifth, donor quality metrics (e.g., KDPI), cold ischemia time, HLA mismatch, and donor-specific antibodies were unavailable or inconsistently recorded in the de-identified dataset and therefore could not be reliably included. Given the importance of these factors in renal transplant, it is possible their absence could result in overweighting of included factors or inadvertent confounding with included variables. Sixth, donor type is widely recognized as an important determinant of kidney allograft outcomes; however, donor status did not retain independent predictive value in our penalized model. In our cohort, living donation was uncommon and similarly distributed between groups (Table 1), limiting the variance and statistical power for donor type to emerge as an independent predictor. In addition, donor-type effects may be partially expressed through ischemia–reperfusion injury and early post-transplant complications (e.g., delayed graft function and rejection) that were not fully captured by the available covariates in this perioperative dataset. Furthermore, it is plausible that donor-type differences are more evident over longer follow-up, and future work with richer donor-quality measures and extended time horizons is needed to better characterize these effects. Finally, the pseudo-R^2^ of 0.21 for our model indicates only modest discrimination; many patients who eventually lost their allograft were not identified by these five factors alone. There remain unmeasured contributors to early failure—such as acute rejection episodes, medication non-adherence, or donor organ injury—that our perioperative variables could not capture. We find it important to note the purpose of the model is hypothesis-generating and intended as a foundation for external validation and refinement before clinical application.

This study lays the groundwork for several future directions. An immediate next step is external validation of the perioperative risk model in other transplant centers or databases. Another important research avenue is to investigate interventions targeting these risk factors. Additionally, the intriguing protective signal for anemia warrants follow-up: a detailed study of pre-transplant anemia management (EPO use, transfusion history, iron status) versus outcomes could clarify whether treating anemia (or how we treat it) influences allograft success. Mechanistic studies may also shed light on how systemic conditions in the recipient modulate allograft resilience. For example, adipose tissue is known to secrete inflammatory cytokines—does this promote immune activation in the allograft? Understanding these pathways could point to targeted therapies (for example, anti-inflammatory strategies for obese recipients). Finally, we see potential in incorporating our perioperative risk factors into multi-factorial prognostic indices alongside traditional markers. As international efforts call for better early surrogates of long-term transplant success, a composite index that includes intraoperative variables (e.g., an “integrated allograft survival score”) could improve early risk stratification and serve as an endpoint in clinical trials of interventions.

## 5. Conclusions

In this single-center perioperative analysis, obesity and thrombocytopenia were associated with substantially increased risk of early allograft failure, whereas coded anemia, female sex, and preoperative clonidine use were associated with lower risk. These findings underscore the potential importance of the recipient’s perioperative profile in shaping early outcomes and highlight several targets for further investigation and external validation. Our findings are in agreement with the literature on enhanced perioperative recovery: they emphasize the need for a holistic, multidisciplinary approach to kidney transplant patients, focusing not just on operative technique and immunosuppression but also on metabolic optimization and hemostatic control [13,25,44]. Ongoing research and validation will determine how to best translate these findings into practice.

## Figures and Tables

**Table 1:** Comparison of demographic and perioperative variables.

Variable	Allograft Failure (n = 53)	No Allograft Failure (n = 266)	*p*-Value
Patient Age	49.17 ± 13.72	51.04 ± 13.64	0.3617
Patient Sex	39.6%	29.6%	0.2204
Donor Status (living) (%)	2.6%	1.9%	1.0000
Length of Stay (days)	7.43 ± 5.34	8.20 ± 13.89	0.6884
Average Post-Operative Pain	2.45 ± 1.56	2.10 ± 1.49	0.1177
Highest Post-Operative Pain	8.54 ± 1.72	8.56 ± 1.91	0.9450
Medical Comorbidities			
Anemia in CKD	39 (73.6%)	150 (56.4%)	0.0218 *
Obesity	12 (21.1%)	196 (73.6%)	<0.0001 *
Thrombocytopenia	8 (14.7%)	90 (34.0%)	0.0017 *
Anemia	38 (67.9%)	65 (24.5%)	<0.0001 *
Relevant Medications			
Dextrose 50% IV	19 (34.6%)	235 (88.7%)	<0.0001 *
Glucagon 1 mg IM	24 (43.6%)	45 (17.0%)	0.0002 *
Acetaminophen	12 (21.8%)	15 (5.7%)	0.0041 *
Midazolam	13 (24.5%)	106 (39.8%)	0.0428 *

* = *p*-value < 0.05. BMI = body mass index, CKD = chronic kidney disease, IM = intramuscular, IV = intravenous.

## Data Availability

The data that support the findings of this study are openly available in the MOVER database at https://mover.ics.uci.edu/index.html (accessed on 7 December 2025), reference [14].

## Supplementary Materials

The following supporting information can be downloaded at https://www.mdpi.com/article/10.3390/healthcare14030341/s1: Table S1: Detailed definitions of all study variables; Table S2: basic statistical analysis for all assessed variables.

## Abbreviations

The following abbreviations are used in this manuscript:

MOVER	Medical Informatics Operating Room Vitals and Events Repository
TRIPOD	Transparent Reporting of a Multivariable Prediction Model for Individual Prognosis or Diagnosis
HIPAA	Health Insurance Portability and Accountability Act
CPT	Current procedural terminology
ICD-9-CM	International classification of diseases codes
ASA	American Society of Anesthesiologists
GLM	Generalized Linear Model
HLA	Human Leukocyte Antigen
GFR	Glomerular Filtration Rate
AKI	Acute Kidney Injury
CKD	Chronic Kidney Disease
ICU	Intensive Care Unit
