# Supplementary material for: Predicting 2-Year Early Allograft Failure After Kidney Transplant: A Perioperative Risk Model from the MOVER Cohort
Source: Healthcare (Basel). 2026 Jan 29;14(3):341. doi: 10.3390/healthcare14030341 (PMC12896626; doi:10.3390/healthcare14030341)
Supplement: Supplementary file 1 [file healthcare-14-00341-s001.zip › healthcare-4063914-supplementary.pdf]

Table S1. Detailed definitions of all study variables

Variable	Allograft Failure (n = 53)
Demographics	
Patient age	Age in years
Patient sex	Patient-reported sex at the time of procedure
Patient BMI	Body mass index (%)
Transplant and Hospitalization Characteristics	
Kidney transplant recipient	ICD-9: V42.0
Donor status (living)	ICD-9: V59.4
Length of stay (days)	Length of hospitalization following transplant (days)
ICU admission	Admission to the intensive care unit during transplant hospitalization
Post-operative Pain Measures	
Average post-operative pain	Patient-reported pain on visual analog scale
Highest post-operative pain	Patient-reported pain on visual analog scale
Past Medical History	
End-stage renal disease	ICD-9: 585.6
Dialysis dependence	ICD-9: V45.1
Anemia in CKD	ICD-9: 285.21
Hypertensive CKD	ICD-9: 403.10, 403.11
Essential hypertension	ICD-9: 401.0, 401.1, 401.9
Hyperlipidemia	ICD-9: 272.4
Type 2 diabetes mellitus with CKD	ICD-9: 250.40, 250.41, 250.43
Type 2 diabetes mellitus	ICD-9: 250.xx
Long-term insulin use	ICD-9: V58.67
Secondary hyperparathyroidism	ICD-9: 588.81
Coronary artery disease	ICD-9: 414.0 and subcodes
Nicotine dependence	ICD-9: 305.1
Obesity	ICD-9: 278.0, 278.01
Gastroesophageal reflux disease	ICD-9: 530.81
Hypothyroidism	ICD-9: 244.9

Hypertensive heart disease with heart failure	ICD-9: 402.1, 402.11, 402.91
COVID-19 exposure	ICD-9: V01.85
COVID-19	ICD-10: U07.1
Acute postprocedural pain	ICD-9: 338.18
Acute post-hemorrhagic anemia	ICD-9: 285.1
Anemia (other)	ICD-9: 285.9
Thrombocytopenia	ICD-9: 287.3–287.5
Other phosphorus metabolism disorders	ICD-9: 275.3
Urinary tract infection	ICD-9: 599.0
Long-term aspirin use	ICD-9: V58.66
Other long-term drug therapy	ICD-9: V58.69
Adverse glucocorticoid effects	ICD-9: E932.0
Metabolic Disturbances (Pre-operative)	
Hypokalemia	ICD-9: 276.8
Hypomagnesemia	ICD-9: 275.2
Hypo-osmolality / hyponatremia	ICD-9: 276.1
Acidosis	ICD-9: 276.2
Hyperkalemia	ICD-9: 276.7
Family History	
Ischemic heart disease / circulatory disorders	ICD-9: V17.3
Diabetes mellitus	ICD-9: V18.0
Peri-operative Factors	
ASA score	American Society of Anesthesiologists physical status classification
Primary anesthesia type	General anesthesia (0) or monitored airway (1)
Surgery duration (min)	Total operative time (minutes)
Pre-operative GFR	Glomerular filtration rate (mL/min/1.73 m^2)
Pre-operative creatinine	Serum creatinine (mg/dL)
Pre-operative hemoglobin	Hemoglobin (g/dL)
Baseline systolic BP	Systolic blood pressure (mmHg)
Baseline diastolic BP	Diastolic blood pressure (mmHg)

Baseline mean arterial pressure	MAP = (2 × diastolic BP + systolic BP)/3
Intra-operative morphine dose	Morphine milligram equivalents (MME) administered intra-operatively
Post-operative morphine dose	MME administered post-operatively
Pressor use	Yes (1) or no (0)
Intra-operative urine output	Urine output during surgery (mL)
Furosemide dose	Intra-operative furosemide (mg)
Average volatile anesthetic (MAC)	Minimum alveolar concentration
Lowest intra-operative MAP (cuff)	Lowest MAP via non-invasive cuff (mmHg)
Lowest intra-operative MAP (arterial line)	Lowest MAP via arterial line (mmHg)
MAP <65 mmHg duration	Minutes with MAP <65 mmHg
MAP <60 mmHg duration	Minutes with MAP <60 mmHg
Average MAP	Average MAP during surgery (mmHg)
Lowest heart rate	Lowest intra-operative heart rate (bpm)
HR >100 bpm duration	Minutes with HR >100 bpm
HR <60 bpm duration	Minutes with HR <60 bpm
HR <50 bpm duration	Minutes with HR <50 bpm
Lowest temperature	Lowest recorded temperature (°C)
Temperature <36 °C duration	Minutes with temperature <36 °C
Temperature <35 °C duration	Minutes with temperature <35 °C
Lowest oxygen saturation	Lowest SpO_2 (%)
SpO_2 <92% duration	Minutes with SpO_2 <92%
Average oxygen saturation	Mean intra-operative SpO_2 (%)
Average exhaled tidal volume	Mean exhaled tidal volume (mL)
Average set tidal volume	Ventilator set tidal volume (mL)
Average PEEP	Positive end-expiratory pressure (cmH_2O)
Average respiratory rate	Mean respiratory rate (breaths/min)
Average set respiratory rate	Ventilator-set respiratory rate (breaths/min)
Average set FiO_2	Fraction of inspired oxygen (%)
Average end-tidal CO_2	End-tidal CO_2 (mmHg)
Post-operative Complications	

Any complication	Any documented post-operative complication (yes/no); low-frequency events (n = 9) consolidated into a single category

Table S2. Basic statistical analysis for all assessed variables

Variable	Allograft Failure (n = 53)	No Allograft Failure (n = 266)	p-value
End-stage renal disease	100.0%	100.0%	1.000
Dialysis dependence	100.0%	100.0%	1.000
ICU admission	100.0%	100.0%	1.000
Anemia	12 (23.1%)	181 (68.0%)	<0.0001
Obesity	39 (75.0%)	56 (21.1%)	<0.0001
Thrombocytopenia	18 (34.6%)	39 (14.7%)	0.0013
Anemia in chronic kidney disease	39 (73.6%)	150 (56.4%)	0.0218
Average post-operative pain	1.6 [1.0, 2.9]	2.2 [1.2, 3.3]	0.0602
Adverse effect of glucocorticoids	11 (21.2%)	31 (11.7%)	0.0741
Other long-term drug therapy	36 (67.9%)	146 (54.9%)	0.0948
Type 2 diabetes mellitus	17 (32.7%)	57 (21.4%)	0.1049
Acidosis	17 (32.7%)	59 (22.2%)	0.1117
Hypothyroidism	10 (19.2%)	30 (11.3%)	0.1153
Hypertensive heart disease with heart failure	10 (19.2%)	30 (11.3%)	0.1153
Female sex	15 (28.3%)	106 (39.8%)	0.1235
Personal history of COVID-19	11 (21.2%)	35 (13.2%)	0.1360
Family history of diabetes mellitus	17 (32.7%)	62 (23.3%)	0.1625
Other phosphorus metabolism disorders	14 (26.9%)	50 (18.8%)	0.1885
Acute post-hemorrhagic anemia	15 (28.8%)	54 (20.3%)	0.1977
Hypo-osmolality / hyponatremia	15 (28.8%)	55 (20.7%)	0.2028
Hypokalemia	12 (23.1%)	43 (16.2%)	0.2325
Long-term aspirin use	13 (25.0%)	46 (17.3%)	0.2404
Gastro-esophageal reflux disease	13 (25.0%)	46 (17.3%)	0.2404
Urinary tract infection	13 (25.0%)	47 (17.7%)	0.2448
Age (years)	53.0 [38.0, 61.0]	50.0 [38.0, 60.0]	0.2645
BMI (kg/m²)	28.2 [22.9, 32.9]	26.7 [24.2, 30.4]	0.4325
ASA score	3.0 [3.0, 3.0]	3.0 [3.0, 3.0]	0.4336

Surgery duration (min)	316.0 [271.0, 345.0]	302.5 [268.0, 336.0]	0.4371
Length of stay (days)	6.0 [5.0, 8.0]	6.0 [5.0, 8.0]	0.5381
Baseline systolic BP	151.0 [135.0, 171.0]	152.0 [135.0, 170.5]	0.8072
Baseline diastolic BP	81.0 [71.0, 92.0]	80.0 [72.0, 88.5]	0.5298
Baseline MAP	102.0 [91.0, 114.0]	102.0 [93.0, 115.0]	0.8014
Lowest intra-operative MAP	40.5 [12.5, 57.8]	39.0 [9.8, 56.0]	0.6880
Average MAP	89.1 [83.8, 95.5]	87.2 [82.1, 92.2]	0.1343
Temperature duration <35 °C (min)	28.0 [2.0, 47.0]	34.0 [7.0, 77.5]	0.0904
Temperature duration <36 °C (min)	222.0 [190.2, 255.0]	215.0 [178.0, 256.0]	0.3462
Average oxygen saturation (%)	99.8 [98.9, 99.9]	99.8 [99.2, 100.0]	0.1882
Lowest oxygen saturation (%)	96.0 [93.0, 97.0]	96.0 [92.0, 98.0]	0.4873
Intra-operative urine output (mL)	70.0 [27.5, 187.0]	62.5 [25.0, 200.0]	0.8225
Intra-operative opioids (MME)	29.0 [20.0, 40.0]	30.0 [20.0, 38.0]	0.9738
Post-operative opioids (MME)	55.8 [30.0, 102.0]	63.2 [30.0, 139.8]	0.3139
Any post-operative complication	2 (3.8%)	7 (2.6%)	0.6478
Dextrose 50% IV solution	52 (98.1%)	128 (48.1%)	<0.0001
Glucagon HCl 1 mg injection	9 (17.0%)	116 (43.6%)	0.0002
Acetaminophen	3 (5.7%)	58 (21.8%)	0.0041
Sodium chloride flush	17 (32.1%)	39 (14.7%)	0.0048
Docusate sodium	3 (5.7%)	1 (0.4%)	0.0154
Midazolam	13 (24.5%)	106 (39.8%)	0.0428
Sodium bicarbonate	2 (3.8%)	1 (0.4%)	0.0728
Prednisone	2 (3.8%)	1 (0.4%)	0.0728
Heparin sodium	2 (3.8%)	2 (0.8%)	0.1301
Metoprolol tartrate	3 (5.7%)	8 (3.0%)	0.4006
Methylprednisolone	21 (39.6%)	129 (48.5%)	0.2916
Insulin regular	1 (1.9%)	1 (0.4%)	0.3051
Furosemide (intra-operative)	100.0 [80.0, 160.0]	100.0 [80.0, 160.0]	0.7254
Tacrolimus	3 (5.7%)	26 (9.8%)	0.4401
Vancomycin	1 (1.9%)	3 (1.1%)	0.5184
Albumin 25% IV	1 (1.9%)	8 (3.0%)	1.000
Ondansetron	1 (1.9%)	4 (1.5%)	1.000
Basiliximab	1 (1.9%)	4 (1.5%)	1.000
Pneumococcal vaccine	1 (1.9%)	5 (1.9%)	1.000
Famotidine	1 (1.9%)	7 (2.6%)	1.000
Cefazolin	1 (1.9%)	7 (2.6%)	1.000

Plasma-Lyte A	6 (11.3%)	43 (16.2%)	0.5308
Senna	2 (3.8%)	8 (3.0%)	0.6743
Amlodipine	2 (3.8%)	8 (3.0%)	0.6743
Labetalol	2 (3.8%)	8 (3.0%)	0.6743
Hydralazine	3 (5.7%)	19 (7.1%)	1.000
Carvedilol	3 (5.7%)	13 (4.9%)	0.7357
